# SPC-Cre-ER^T2^ Transgenic Mouse for Temporal Gene Deletion in Alveolar Epithelial Cells

**DOI:** 10.1371/journal.pone.0046076

**Published:** 2012-09-25

**Authors:** Yao-Song Gui, Lianmei Wang, Xinlun Tian, Ruie Feng, Aiping Ma, Baiqiang Cai, Hongbing Zhang, Kai-Feng Xu

**Affiliations:** 1 Department of Respiratory Medicine, Peking Union Medical College Hospital, Peking Union Medical College and Chinese Academy of Medical Sciences, Beijing, China; 2 State Key Laboratory of Medical Molecular Biology, Department of Physiology and Pathophysiology, Institute of Basic Medical Sciences, Peking Union Medical College and Chinese Academy of Medical Sciences, Beijing, China; 3 Department of Pathology, Peking Union Medical College Hospital, Peking Union Medical College and Chinese Academy of Medical Sciences, Beijing, China; National University of Singapore, Singapore

## Abstract

Although several Cre-loxP-based gene knockout mouse models have been generated for the study of gene function in alveolar epithelia in the lung, their applications are still limited. In this study, we developed a SPC-Cre-ER^T2^ mouse model, in which a tamoxifen-inducible Cre recombinase (Cre-ER^T2^) is under the control of the human surfactant protein C (SPC) promoter. The specificity and efficiency of Cre-ER^T2^ activity was first evaluated by crossing SPC-Cre-ER^T2^ mouse with ROSA26R mouse, a β-galactosidase reporter strain. We found that Cre-ER^T2^ was expressed in 30.7% type II alveolar epithelial cells of SPC-Cre-ER^T2^/ROSA26R mouse lung tissues in the presence of tamoxifen. We then tested the tamoxifen-inducible recombinase activity of Cre-ER^T2^ in a mouse strain bearing *TSC1* conditional knockout alleles (TSC1^fx/fx^). *TSC1* deletion was detected in the lungs of tamoxifen treated SPC-Cre-ER^T2^/TSC1^fx/fx^ mice. Therefore this SPC-Cre-ER^T2^ mouse model may be a valuable tool to investigate functions of genes in lung development, physiology and disease.

## Introduction

Gene knockout technology has been widely used in mice to determine the functions of a specific gene in mouse development and its contribution to a particular disease. Conventional gene knockout ablates a gene in all cells, usually resulting in complex phenotypes, or embryonic lethality if the gene product is critical in development. Therefore, conditional or tissue-specific gene knockout strategies have been developed. One of these strategies is using a Cre-loxP recombination system. This system consists of a 38 kD Cre DNA recombinase and two 34 bp loxP sites flanking a target DNA sequence. The Cre recombinase recognizes loxP sites and excises the target DNA sequence when the orientation of two loxP sites is cis-repeated [1]. For a tissue-specific gene knockout strategy, the Cre coding sequence is usually driven by a tissue-specific promoter, which allows Cre to be expressed only in one type of tissue. Thus, deletion of the gene of interest also occurs only in the tissue where Cre is expressed.

Using Cre/loxP system to specifically knock out genes in alveolar epithelial cells has been reported by several research groups [2], [3]. In these reported Cre transgenic mouse models, a surfactant protein C (SPC) promoter is used to drive Cre expression. The surfactant protein C is exclusively expressed in the type II alveolar epithelial cells [4]. A SPC-Cre transgenic mouse was generated to understand the role of VEGF in alveolar structure, acute inflammation, and vascular permeability [2]. In this model, the Cre-mediated recombination begins at embryo period and is not controlled. This model is useful to study lung development at embryonic stage, but has limitations for study at adult stage.

To prevent the possible embryonic lethality caused by gene deletion in early embryo development, a SPC-rtTA/TetO-Cre transgenic mouse model has been developed [3]. In this model, Cre-recombinase activity can be controlled by doxycycline temporally. However, the breeding scheme for getting three transgenes in one mouse is very complicated and time-consuming [3]. In addition, the rtTA toxicity in SPC-rtTA mice leads to impaired alveologenesis, abnormal expression of surfactant-associated proteins, and mouse death, which limits the use of this model in research [5], [6].

An inducible Cre-recombinase, Cre-ER^T2^, has been developed by fusing the Cre coding sequence with a mutant form of ligand-binding domain of the estrogen receptor (ER^T2^). The Cre-ER^T2^ is active only in the presence of tamoxifen. A number of tissue-specific Cre-ER^T2^ mouse models have been reported [7], [8], [9]. In this study, we developed a novel SPC-Cre-ER^T2^ mouse model which can be applied for gene knock-out in mouse alveolar epithelium in a temporally controlled fashion. We tested this model in both ROSA26R and TSC1^fx/fx^ transgenic mice.

## Results

### Generation of the SPC-Cre-ER^T2^ Transgenic Mice

**Figure 1:**
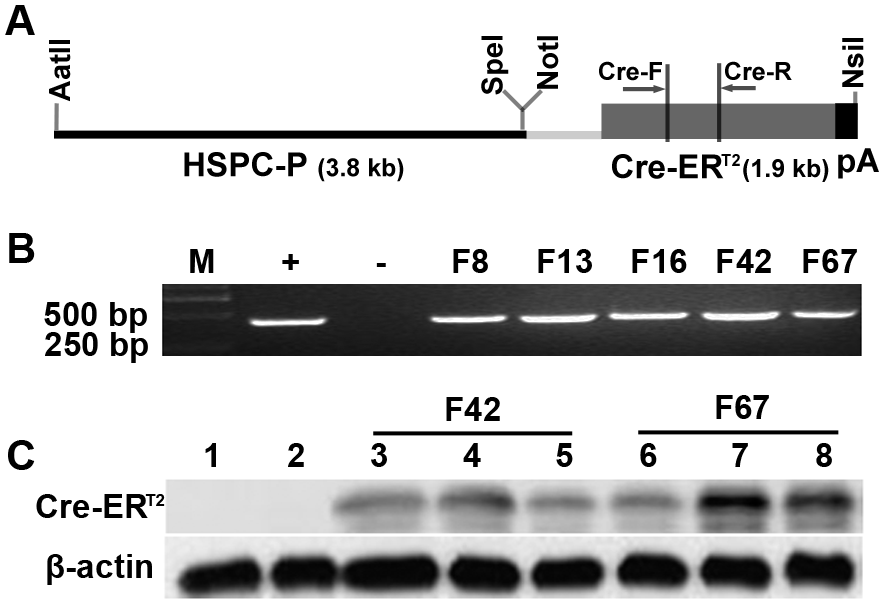
Generation of SPC-Cre-ER^T2^ mice. A) Schematic map of SPC-Cre-ER^T2^ expression cassette. HSPC-P, human surfactant protein C promoter; Cre-ER^T2^, Cre coding sequence fused with a tamoxifen-inducible estrogen receptor. pA, a polyA sequence from SV40 virus. Cre-F primer binding sites, 561–579 bp of Cre-ER^T2^ transgene; Cre-R primer binding sites, 976–997 bp of Cre-ER^T2^ transgene. The map is drawn in scale. B) Screening SPC-Cre-ER^T2^ transgenic mice using PCR. Genomic DNA from each mouse tail was used as template to specifically PCR-amplify the Cre-ER^T2^ transgene. M, DNA marker; +, SPC-Cre-ER^T2^ plasmid DNA control; -, water control; F8, F13, F16, F42, F67 are five representative founder transgenic mice generated by microinjection of SPC-Cre-ER^T2^ expression cassette into fertilized embryos. C) Cre-ER^T2^ fusion proteins were detected using Western blot in lung tissues of SPC-Cre-ER^T2^ transgenic mice. Lane 1, C57BL/6J mouse; Lane 2, an offspring of F42 founder without Cre-ER^T2^ transgene when genotyped using PCR; Lane 3, 4, 5, offspring of F42 founder; Lane 6, 7, 8, offspring of F67 founder. Notice the variable expression levels of Cre-ER^T2^ in offspring from the same founder.

A 9.5 kb AatII-NsiI DNA fragment containing SPC-Cre-ER^T2^ expression cassette (Fig. 1A) was microinjected into embryos of C57BL/6J mice. These embryos were transferred into pseudo-pregnant surrogate C57BL/6J mice to obtain pups. We used PCR to screen for mice bearing SPC-Cre-ER^T2^ and found five mice positive for Cre-ER^T2^ (Fig. 1B). These five founders were bred with C57BL/6J mice to transmit SPC-Cre-ER^T2^ transgene.

### Cre-ER^T2^ Expression in Lung Tissues

We then asked whether Cre-ER^T2^ fusion proteins were stably expressed in the lung tissues of the offspring of these five founders. Cre-ER^T2^ fusion proteins in the lung tissues of 4-week-old mice were analyzed using Western blot with monoclonal antibody against Cre. We found that Cre-ER^T2^ was expressed stably only in the offspring from two of these five founders (Fig. 1C). Therefore, the offspring from these two founders (F42 and F67) were used for the following experiments.

### Cre-ER^T2^ Recombinase Activity in SPC-Cre-ER^T2^/ROSA26R Transgenic Mice

**Figure 2:**
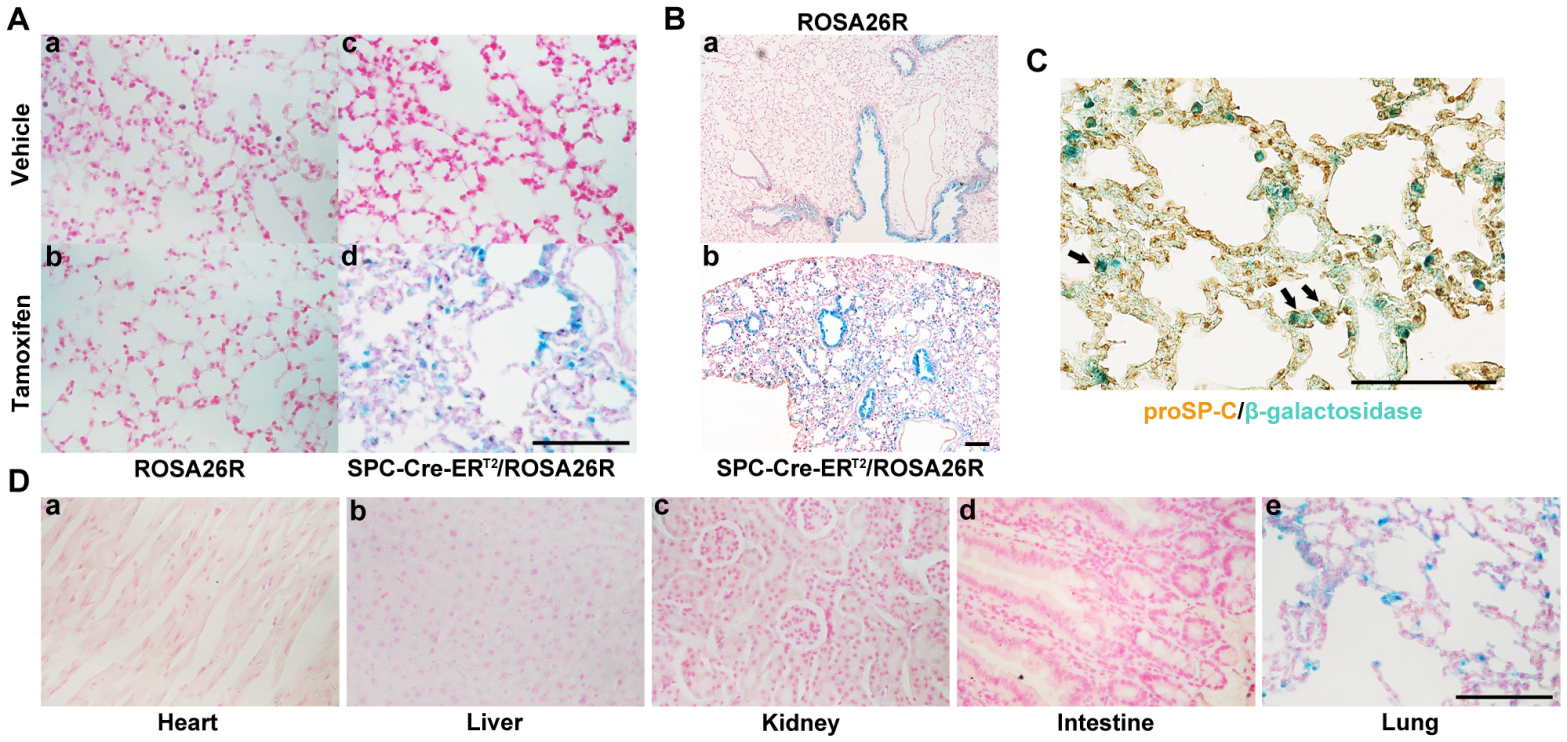
Tamoxifen-inducible and tissue-specific recombinase activity of Cre-ER^T2^ in SPC-Cre-ER^T2^/ROSA26R transgenic mice. A) β-galactosidase activity was detected in the lung alveolar epithelia from a SPC-Cre-ER^T2^/ROSA26R transgenic mouse receiving tamoxifen treatment. a & b, lung tissue sections from ROSA26R mice (without Cre-ER^T2^ transgene) receiving vehicle (a) or tamoxifen (b); c & d, lung tissue sections from SPC-Cre-ER^T2^/ROSA26R transgenic mice receiving vehicle (c) or tamoxifen (d). B) Endogenous β-galactosidase activity was found in lung bronchial epithelia of ROSA26R mouse receiving vehicle (a) and SPC-Cre-ER^T2^/ROSA26R transgenic mouse after tamoxifen treatment (b). C) X-gal stained lung tissues of SPC-Cre-ER^T2^/ROSA26R transgenic mouse receiving tamoxifen were also immune-stained for proSP-C, an alveolar type II cell-specific marker. Arrows indicate three representative alveolar type II cells co-expressing proSP-C (brown) and β-galactosidase (blue). D) β-galactosidase activity was detected only in the lung alveolar epithelium, but not in other organs from a SPC-Cre-ER^T2^/ROSA26R transgenic mouse receiving tamoxifen treatment. a, heart; b, liver; c, kidney; d, intestine; e, lung. All scale bars in this figure equal 100 µm.

To test fusion protein, we asked whether Cre-ER^T2^ fusion protein could turn on *LacZ* gene expression in ROSA26R mice [10] in the presence of tamoxifen. The SPC-Cre-ER^T2^ mice were bred with ROSA26R mice to get SPC-Cre-ER^T2^/ROSA26R mice. The β-galactosidase activity was detected only in the alveolar epithelial cells of those mice receiving tamoxifen, but not vehicle, suggesting that the Cre-ER^T2^ fusion protein was functional in the alveolar epithelial cells of mice in the presence of tamoxifen (Fig. 2A).

We then asked the tamoxifen-induction efficiency of the Cre-ER^T2^ in type II alveolar epithelial cells by using immunohistochemical staining against alveolar type II cell-specific marker protein, proSP-C, and X-gal staining in the lung tissues of 4-week-old SPC-Cre-ER^T2^/ROSA26R mice receiving tamoxifen (1 mg/day) for five consecutive days. About 30.7% alveolar type II epithelial cells were β-galactosidase positive (Fig. 2C).

We also investigated the specificity of the SPC-Cre-ER^T2^ in the lung tissues of those SPC-Cre-ER^T2^/ROSA26R mice. Endogenous β-galactosidase staining was found in bronchial epithelia from ROSA26R mice receiving vehicle and SPC-Cre-ER^T2^/ROSA26R mice with tamoxifen treatment (Fig. 2B). We found that, in lung tissue, excluding bronchial epithelial cells, 51.8% of β-galactosidase positive cells were alveolar type II cells (Fig. 2C). Furthermore, the tamoxifen-induced recombinase activity of Cre-ER^T2^ existed only in lung, but not in heart, liver, kidney, and intestine, suggesting that the SPC promoter conveyed tissue-specific activity in the SPC-Cre-ER^T2^ transgene (Fig. 2D).

### Cre-ER^T2^ Recombinase Activity in SPC-Cre-ER^T2^/TSC1^fx/fx^ Transgenic Mice

**Figure 3:**
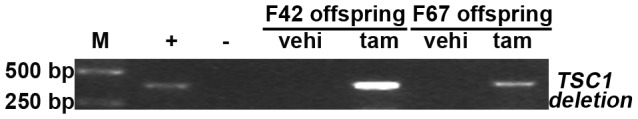
Tamoxifen-induced recombinase activity of Cre-ER^T2^ in the lung tissues of SPC-Cre-ER^T2^/TSC1^fx/fx^ transgenic mice. DNAs from the lung tissues of SPC-Cre-ER^T2^/TSC1^fx/fx^ transgenic mice treated with vehicle or tamoxifen were examined by PCR to detect *TSC1* deletion as an indication of Cre-ER^T2^ recombinase activity. M, DNA marker; +, a transgenic mouse with *TSC1* deletion; -, C57BL/6J mouse. Other lanes, offspring from F42 and F67 founders treated with vehicle (vehi) or tamoxifen (tam).

We also tested the tamoxifen-inducible recombinase activity of Cre-ER^T2^ in the TSC1^fx/fx^ transgenic mice [11]. The SPC-Cre-ER^T2^/TSC1^fx/fx^ mice were obtained by breeding SPC-Cre-ER^T2^ with TSC1^fx/fx^ mice as described in the section of materials and methods. After given tamoxifen or vehicle, the SPC-Cre-ER^T2^/TSC1^fx/fx^ mice were sacrificed, their lung tissues were harvested, and their genomic DNAs were extracted to detect *TSC1* deletion by PCR [11]. We found that *TSC1* deletion was detected only in mice treated with tamoxifen, but not vehicle, suggesting that the Cre-ER^T2^ exerted its recombinase activity only in the presence of tamoxifen (Fig. 3).

## Discussion

In this study, we generated a SPC-Cre-ER^T2^ transgenic mouse model which can be used to study the functions of genes in mouse alveolar epithelial cells in any stage of mouse development. We tested this model in two commonly used transgenic mouse strains carrying either ROSA26R [10] or *TSC1* conditional knockout alleles (TSC1^fx/fx^) [11] and found that this novel transgenic mouse model expressed Cre-ER^T2^ fusion proteins predominately in alveolar epithelial cells and Cre recombinase activity was active only in the presence of tamoxifen (Fig. 2 and 3).

Our SPC-Cre-ER^T2^ transgenic mouse model has several advantages when compared with other transgenic mouse models which have been used for conditionally knocking out genes for lung development and functions [2], [3]. First, in our transgenic mouse model we can control when Cre recombination takes place by tamoxifen administration, which allows scientists to knock out the gene of interest in a temporally controlled manner. Second, using single Cre-ER^T2^ transgene in our transgenic mouse model makes the subsequent mouse breeding process simpler than using two transgenes in the SPC-rtTA/TetO-Cre mouse model [3].

We observed variable Cre-ER^T2^ expression in our SPC-Cre-ER^T2^ transgenic mice. Three out of five SPC-Cre-ER^T2^ founders could not give birth of mice expressing Cre-ER^T2^ in lungs. Furthermore, the Cre-ER^T2^ expression levels in the lungs of offspring from the same founders were variable (Fig. 1C). We also observed the different degree of β-galactosidase staining in the lungs of mice from the same founder line. One possible reason for the difference of transgene expression levels in offspring from the same founder line might be that multiple integration sites occurred in the founder mice.

In a healthy human lung, 15% cells are alveolar type II epithelial cells [12]. In our study, an average of 30.7% alveolar type II cells in the lungs of the SPC-Cre-ER^T2^/ROSA26R mice treated with tamoxifen expressed β-galactosidase. This may be due to the lack of an optimal condition for inducing Cre-ER^T2^ by tamoxifen in our transgenic mice. The optimal conditions for inducing gene inactivation by administrating tamoxifen are different in different mouse models [7], [8], [9], [13], [14]. An optimal condition per mouse model can be affected by the following factors, the means of drug administration, the dose of tamoxifen, and the age, gender, and genetic background of the experimental mice. Further studies to determine an optimal tamoxifen-induction protocol for our transgenic mouse model are necessary for its wide application.

Another discrepancy in our study was that only 51.8% β-galactosidase positive cells (excluding the β-galactosidase positive bronchial epithelia) in lung tissues of SPC-Cre-ER^T2^/ROSA26R mice receiving tamoxifen were alveolar type II cells (Fig. 2C). Other β-galactosidase positive cells were bronchial epithelial cells (Fig. 2B–b) and a few of other types of cells. The β-galactosidase positive cells in bronchial epithelium were also observed in ROSA26R mice (Fig. 2B-a) or SPC-Cre-ER^T2^/ROSA26R mice without tamoxifen treatment (data not shown). Thus, the β-galactosidase activity in bronchial epithelium is caused by its endogenous activity. A few cells which were β-galactosidase positive and proSP-C negative were observed in the lung tissues of our experimental mice. We did not further clarify these cells. Some of them might be alveolar type I cells because of the alveolar type II cells to type I cells transdifferentiation [15].

In summary, we generated a novel transgenic mouse model, SPC-Cre-ER^T2^ mice, which express tamoxifen-inducible Cre recombinase in alveolar epithelial cells. This mouse model can be used to conditionally knock out genes in alveolar epithelial cells at any stages of lung development. Therefore, it will be a useful tool in understanding gene functions in lung development, physiology and disease.

## Materials and Methods

### Ethics Statement

All animal experiments were approved by the Animal Ethics Committee of Peking Union Medical College according to international and institutional guidelines for animal care. All surgery was performed under sodium pentobarbital anesthesia, and all efforts were made to minimize suffering.

### Plasmid and Transgene Construction

A SPC-Cre-ER^T2^ expression construct (Fig. 1A) was generated as following steps. First, a 3.8-kb AatII/SpeI human surfactant protein C promoter (HSPC-P) from pUC-SPC plasmid (a gift from Dr. Jeffrey A. Whitsett, Cincinnati Children’s Hospital Medical Center) was cloned to a pGEM-TEasy vector (Promega, USA) to generate pGEM-SPC. Then, a 2.8-kb NotI/NsiI fragment containing Cre-ER^T2^ cDNA and the polyadenylation site (poly A) from the SV40 early region from a pCAG-Cre-ER^T2^ (Addgene, USA) [16] was cloned to the pGEM-SPC to get pGEM-SPC-Cre-ER^T2^ (Fig. 1A).

### Generation of Transgenic Mice

To generate SPC-Cre-ER^T2^ transgenic mice, a 9.5-kb AatII/NsiI fragment from pGEM-SPC-Cre-ER^T2^ construct was microinjected into fertilized embryos of C57BL/6J mice. The SPC-Cre-ER^T2^ founder lines were bred with C57BL/6J wild type mice to generate the SPC-Cre-ER^T2^ F1 mice. Offspring were genotyped as described below. Mice were maintained in a pathogen-free mouse facility.

### Western Blot Analysis for Cre-ER^T2^ Expression

Total proteins were extracted from mouse lung tissues and separated on a 12% SDS-PAGE gel. The separated proteins were then transferred onto a PVDF membranes (Pierce, USA) for detecting Cre-ER^T2^ and β-actin proteins with monoclonal antibodies against Cre (1∶1,000, Covance, USA) and β-actin (1∶1,000, Santa Cruz, USA), respectively. The β-actin expression was used as a loading control of protein extracts.

### Analysis of Cre-ER^T2^ Activity

We analyzed Cre-ER^T2^ recombinase activity in two transgenic mouse models: ROSA26R and TSC1^fx/fx^ mice. ROSA26R mouse strain bear a lox-STOP-lox-cassette inserted between a promoter and a *LacZ* gene [10] and TSC1^fx/fx^ mice bear loxP-flanked *TSC1* conditional knockout alleles [11]. The SPC-Cre-ER^T2^/ROSA26R mice were obtained via breeding SPC-Cre-ER^T2^ mice with homozygous ROSA26R mice. The SPC-Cre-ER^T2^/TSC1^fx/fx^ mice were obtained via two steps of breeding. First, the SPC-Cre-ER^T2^ transgenic mice were bred with TSC1^fx/fx^ mice to get SPC-Cre-ER^T2^/TSC1^fx/+^ mice. Then these SPC-Cre-ER^T2^/TSC1^fx/+^ mice were bred with TSC1^fx/fx^ mice to get SPC-Cre-ER^T2^/TSC1^fx/fx^ mice. All transgenic mice were genotyped using PCR. Primers used for amplifying Cre transgene were forward primer, 5′-TACTGACGGTGGGAGAATG-3′ and reverse primer, 5′-CTGTTTCACTATCCAGGTTACG-3′. The expected PCR product was 437 bp in size. Primers used for amplifying ROSA26R were forward primer, 5′-GACACCAGACCAACTGGTAATGGTAGCGAC-3′ and reverse primer, 5′-GCATTGAGCTGGGTAATAAGCGTTGGCAAT-3′. The expected PCR product was 750 bp in size. The TSC1^fx/fx^ conditional allele was tested using PCR as previously described [11].

Cre-ER^T2^ recombinase activity in these transgenic mice was induced by tamoxifen [9], [14]. Briefly, tamoxifen (Sigma, USA) was dissolved in a sunflower seed oil/ethanol mixture (10∶1) at 10 mg/ml. Each 4-week-old mouse was injected intraperitoneally with 100 µl of tamoxifen per day for 5 consecutive days. One week after the last injection, mice were sacrificed and the lungs, hearts, livers, kidneys, intestines of the SPC-Cre-ER^T2^/ROSA26R mice were harvested to examine the β-galactosidase activity by X-gal staining and the lungs of SPC-Cre-ER^T2^/TSC1^fx/fx^ mice were harvested to detect the *TSC1* deletion.

For β-galactosidase activity analysis, tissues were fixed in 0.2% glutaraldehyde, 2% formaldehyde in PBS for overnight at 4°C. After washed three times for 45 min with PBS at room temperature with gentle shaking, tissue sections were stained overnight in freshly prepared X-gal staining solution containing 2 mM MgCl_2_, 2.5 mM K_3_Fe(CN)_6_, 2.5 mM K_4_Fe(CN)_6_ and 1 mg/ml X-gal. X-gal stock-solution was prepared in dimethylformamide at 40 mg/ml. Stained tissues were further paraffin embedded and sections were counterstained with nuclear fast red.

To investigate the Cre-ER^T2^-mediated excision efficiency in the lung tissues, we performed immunohistochemical staining for pro-Surfactant Protein C (proSP-C), a specific alveolar type II cell marker and β-galactosidase staining at the same lung tissues. Briefly, X-gal stained lung tissue sections (3 µm) were deparaffinized and rehydrated in xylene, heated in 10 mM citrate buffer, treated with 3% H_2_O_2_ for 10 minutes. After blocked with 5% normal goat serum, these tissue sections were incubated with rabbit-derived primary antibody (anti-mouse proSP-C, 1∶1000, Millipore, USA) at 4°C for overnight followed by incubation with secondary antibody for 1 hour at room temperature. The bound secondary antibodies were detected with VECTASTAIN ABC kit (Vector, USA). Images were taken with Nikon Eclipse 80i microscope (Tokyo, Japan) with a digital camera. The numbers of cells positive for both β-galactosidase and proSP-C, β-galactosidase only, or proSP-C only were obtained respectively by counting total 12 fields with 400×magnification.

For detection of *TSC1* deletion, genomic DNAs were isolated from lung tissues of SPC-Cre-ER^T2^/TSC1^fx/fx^ mice treated with tamoxifen. The *TSC1* deletion was detected using PCR. The primers for *TSC1* deletion were: F4536∶5′-AGGAGGCCTCTTCTGCTACC-3′ and R6548∶5′-TGGGTCCTGACCTATCTCCTA-3′ [11]. The expected PCR product size was 370 bp with excision of exons 17 and 18 of *TSC1* gene [11].
